# Based on Network Pharmacology Tools to Investigate the Mechanism of *Tripterygium wilfordii* Against IgA Nephropathy

**DOI:** 10.3389/fmed.2021.794962

**Published:** 2021-12-15

**Authors:** Ming Xia, Di Liu, Haiyang Liu, Juanyong Zhao, Chengyuan Tang, Guochun Chen, Yu Liu, Hong Liu

**Affiliations:** Hunan Key Laboratory of Kidney Disease and Blood Purification, Department of Nephrology, The Second Xiangya Hospital, Central South University, Changsha, China

**Keywords:** *Tripterygium wilfordii* Hook F, IgA nephropathy, network pharmacology, JUN, mesangial cell

## Abstract

**Background:** Immunoglobulin A nephropathy (IgAN) is the most common primary glomerular disease and poses a global major public health burden. The preparation of *Tripterygium wilfordii* Hook F (TwHF) is widely applied for treating patients with Immunoglobulin A nephropathy in China, while the molecular mechanisms remain unclear. This study aimed to verify the therapeutic mechanism of TwHF on IgAN by undertaking a holistic network pharmacology strategy in combination with *in vitro* and *in vivo* experiments.

**Methods:** TwHF active ingredients and their targets were obtained *via* the Traditional Chinese Medicine Systems Pharmacology Database. The collection of IgAN-related target genes was collected from GeneCards and OMIM. TwHF-IgAN common targets were integrated and visualized by Cytoscape. Gene ontology (GO) and Kyoto Encyclopedia of Genes and Genomes (KEGG) analyses were performed to determine the predominant molecular mechanisms and pathways of TwHF on the treatment of IgAN. The protein-protein interaction network was constructed by the STRING online search tool, and hub genes were identified using R software. The expression of hub gene and related signaling were evaluated in TwHF-treated mice through immunohistochemistry and western blot and further validated in human mesangial cells (HMCs). In addition, Cell counting kit 8 (CCK8) and flow cytometry were used to detect the effects of TwHF on cell proliferation and cell cycle of mesangial cells.

**Results:** A total of 51 active ingredients were screened from TwHF and 61 overlapping targets related to IgAN were considered potential therapeutic targets, GO functions and KEGG analyses demonstrated that these genes were primarily associated with DNA-binding transcription factor binding, lipid and atherosclerosis pathway. Genes with higher degrees including *AKT1, CXCL8, MMP9, PTGS2, CASP3, JUN* are hub genes of TwHF against IgAN. Verification of hub gene JUN both *in vitro* and *in vivo* showed that TwHF significantly attenuated JUN phosphorylation in the kidneys of IgAN mice and aIgA1-activated HMCs, meanwhile suppressing HMCs proliferation and arresting G1-S cell cycle progression.

**Conclusion:** Our research strengthened the mechanisms of TwHF in treating IgAN, inhibition of JUN activation may play a pivotal role in TwHF in alleviating IgAN renal injury.

## Introduction

Immunoglobulin A nephropathy (IgAN) is the most common primary glomerular disease worldwide, especially in the Asia-Pacific region, the proportion is up to 50% of primary glomerular disease in China (1–3). It is characterized by Immunoglobulin A (IgA) deposition in the glomerular mesangial region accompanied by mesangial cells proliferation and matrix expansion. Clinical manifestations are microscopic or gross hematuria, with or without various degrees of proteinuria, elevated blood pressure, and edema. About 20–40% of patients progress to end-stage renal disease (ESRD) within 20 years after diagnosis, bringing a heavy burden to the individual and the whole society (4). “Multi-hits” theory is considered to be the possible pathogenesis: the overproduction of abnormal glycosylated IgA1; autoantibodies against abnormal IgA1; IgA1 immune complexes deposited in the mesangial area; stimulation of complement, cytokines, and immune-inflammatory response leading to kidney damage (5). While there is still a lack of clear understanding of the pathogenesis of IgA nephropathy (IgAN) and short of effective therapeutics for disease progression.

*Tripterygium wilfordii* Hook F (TwHF) is the dried root of the genus *Tripterygium wilfordii* in the euonymus family, which has been used in traditional Chinese medicine for more than 2,000 years (6). As early as 1977, Professor Leishi Li used TwHF to treat various types of glomerulonephritis including IgA nephropathy, and confirmed for the first time that TwHF was beneficial for reducing haematuria and eliminating edema and gradually applied to kidney injury (7). Modern pharmaceutical technology has extracted a variety of biological components from TwHF including alkaloids, diterpenes, triterpenes, sesquiterpenes, and polysaccharides. Among them, diterpene alkaloids such as triptolide and triterpenes are the main active components with immunosuppressive, anti-inflammatory, and anti-tumor effects (8), and their preparation Tripterygium glycoside tablets (TGT) has accumulated a great deal of clinical experience in the treatment of IgAN and has shown efficiency in reducing urine protein and serum creatinine levels (9–11). However, there is still no satisfactory explanation for its pharmacological mechanism related to IgAN.

Network pharmacology is used to identify the interactive network of “compound-targets-disease,” which owns the ability to elucidate complexities for multi-components, multi-targets, and multi pathways from a systemic perspective (12, 13). Thus, we aimed to analyze the pharmacological mechanisms of TwHF involved in IgAN employing a network pharmacology approach combined with experimental research, laying a stable foundation for exploring pharmacological mechanisms of TwHF in treating IgAN.

## Materials and Methods

### Recognition of TwHF Active Ingredients and Correlated Targets

Traditional Chinese Medicine Systems Pharmacology Database (TCMSP, http://tcmspw.com/tcmsp.php, Lab of Systems Pharmacology, China), a unique systematic pharmacology platform that contains the relationships among drugs, targets, and diseases, was applied to identify the chemical constituents of TwHF. The ingredients with oral bioavailability (OB) ≥ 30% and the drug-likeness (DL) ≥0.18 (a suggested criterion of TCMSP database) were selected as active components for further analysis (14). Meanwhile, the target associated with active ingredients of TwHF were gathered based on the TCMSP database and further verified for official target names in the UniProt database (https://www.uniprot.org, UniProt Consortium, UK/USA/Switzerland) (15).

### IgAN Targets Identification and TwHF-IgAN Targets Network Construction

GeneCards (https://www.genecards.org, LifeMap Sciences, USA) and Online Mendelian Inheritance in Man (OMIM, https://www.omim.org, Johns Hopkins University, USA) were used to search for IgAN-related targets, both of which are authoritative human gene compendium web server about genome, transcriptome, disease, and function (16, 17). The overlapping targets of TwHF ingredients and IgAN were obtained using the VennDiagram R package (bioconductor, USA), and the TwHF-IgAN targets network was visualized *via* Cytoscape software (version 3.7.2, https://www.cytoscape.org/, The Cytoscape Consoritum, USA).

### GO and KEGG Pathway Enrichment Analyses

Gene ontology (GO) enrichment and Kyoto Encyclopedia of Genes and Genomes (KEGG) pathway analyses were used to explore the potential biological processes and utilities, cellular components, and molecular functions of overlapped targets. Significant relevant signals were identified *via* clusterProfiler package (bioconductor, USA) in R software (× 64 4.1.0) with *p* < 0.05 and *q* < 0.05. A protein-protein interaction (PPI) network of overlapped target genes was built based on the STRING database (https://cn.string-db.org/, STRING CONSORTIUM, UK/Switzerland/Denmark) (18). Densely connected clusters in the PPI network were visualized by R.

### Experimental Animals and Cells

Female BALB/c mice were obtained from Hunan SJA Laboratory Animal Co. Ltd. (Changsha, China) and housed under pathogen-free conditions, the IgAN mouse model was induced by oral mucosal immune as previously described (19). At 11 weeks of age, mice were randomly assigned to receive TGT gavage (7 mg/kg dissolved in saline) or PBS for 6 weeks. All experiments were performed following the animal experimental guidelines issued by the Animal Care and Use Committee at the Xiangya Medical School of Central South University.

The human mesangial cells (HMCs) (Cellbio, China) were cultivated in Dulbecco's Modified Eagle's Medium (DMEM)/F-12 medium (Gibco, USA) supplemented with 10% fetal bovine serum (FBS) under 5% CO_2_ at 37°C. Monomeric human IgA1 (Abcam, ab91020) was heated and aggregated at 65°C for 150 min on a dry plate heater to obtain aIgA1 as previously described (20). Triptolide was purchased from ApexBio Technology (Houston, USA). Cells were incubated with aIgA1 alone or with a combination of triptolide (0–75 nM) concentrations for 24 h.

### Immunohistochemistry Staining

Formaldehyde-fixed and paraffin-embedded tissue sections (4 mm thick) were used for immunohistochemistry as previously described (21). After dehydration, slides in citrate solution were subjected to a microwave antigen retrieval process. Primary antibodies were employed against the JUN (9165s, Cell Signaling Technology, USA), p-JUN (3270s, Cell Signaling Technology, USA), with periodic acid-Schiff (PAS) used instead of primary antibodies as negative controls for staining. Finally, horseradish peroxidase (HRP)-conjugated polymer (Abcam, USA) was used for the visualized detection under light microscopy (Nikon Tokyo, Japan). OD values were analyzed by ImageJ software (Media Cybernetics, USA). All the analyses were repeated no less than three times, and representative images are displayed. The use of renal specimens of IgAN patients was approved by the Ethics Committee of the Second Xiangya Hospital of Central South University according to the Declaration of Helsinki.

### Western Blot Analysis

Cells and renal tissues were lysed in RIPA Lysis buffer (Beyotime Biotechnology, China) supplemented with PMSF, and protein concentration was determined by BCA assay (Thermo Fisher Scientific, USA). Proteins were subjected to 10% SDS-PAGE and transferred to PVDF membranes (Millipore, USA). The membranes were blocked with 5% bovine serum albumin (BSA) at room temperature for 1 h, and incubated overnight at 4°C with the primary antibodies: JUN (9165s, Cell Signaling Technology, USA), p-JUN (3270s, Cell Signaling Technology, USA), β-actin (GB11001, Servicebio, China). Secondary antibodies were used and visualized by enhanced chemiluminescence (Millipore, USA).

### Cell Proliferation and Cell Cycle Analysis

Cell proliferation was accessed by Cell Counting Kit-8 (CCK8) assay (Dojindo, Japan) following the manufacturer's recommended procedure. In brief, 10 μl of CCK8 was added to treated cells in 96-wells and incubated at 37°C. The absorbance was detected at 450 nm using a Spectrophotometer (Molecular Devices, USA).

Cell cycle was measured by Cell Cycle and Apoptosis Kit (Wellbiology, China), and the treated cells were fixed overnight with 70% cold ethanol at 4°C, then stained with a mixture of propidium iodide and RNase A at 37°C for 30 min. The distribution of the cell cycle phase was measured by FlowJo software version 7.6.1 (Tree Star, USA).

### Statistical Analysis

Statistical analysis was performed in R version. 4.1.0 (The R Foundation) and Graphed Prism 6 (GraphPad Software Inc., USA). Benjamini–Hochberg multiple testing correction was used to calculate the adjusted *p*-value or q-value. The data from *in vitro* and *ex vivo* experiments analyzed using Student's *t*-test and were presented as the mean ± *SD*. For all statistical analyses, *p*-value < 0.05 was considered significant.

## Results

### Common Targets of TwHF Active Ingredients and IgAN

**Figure 1:**
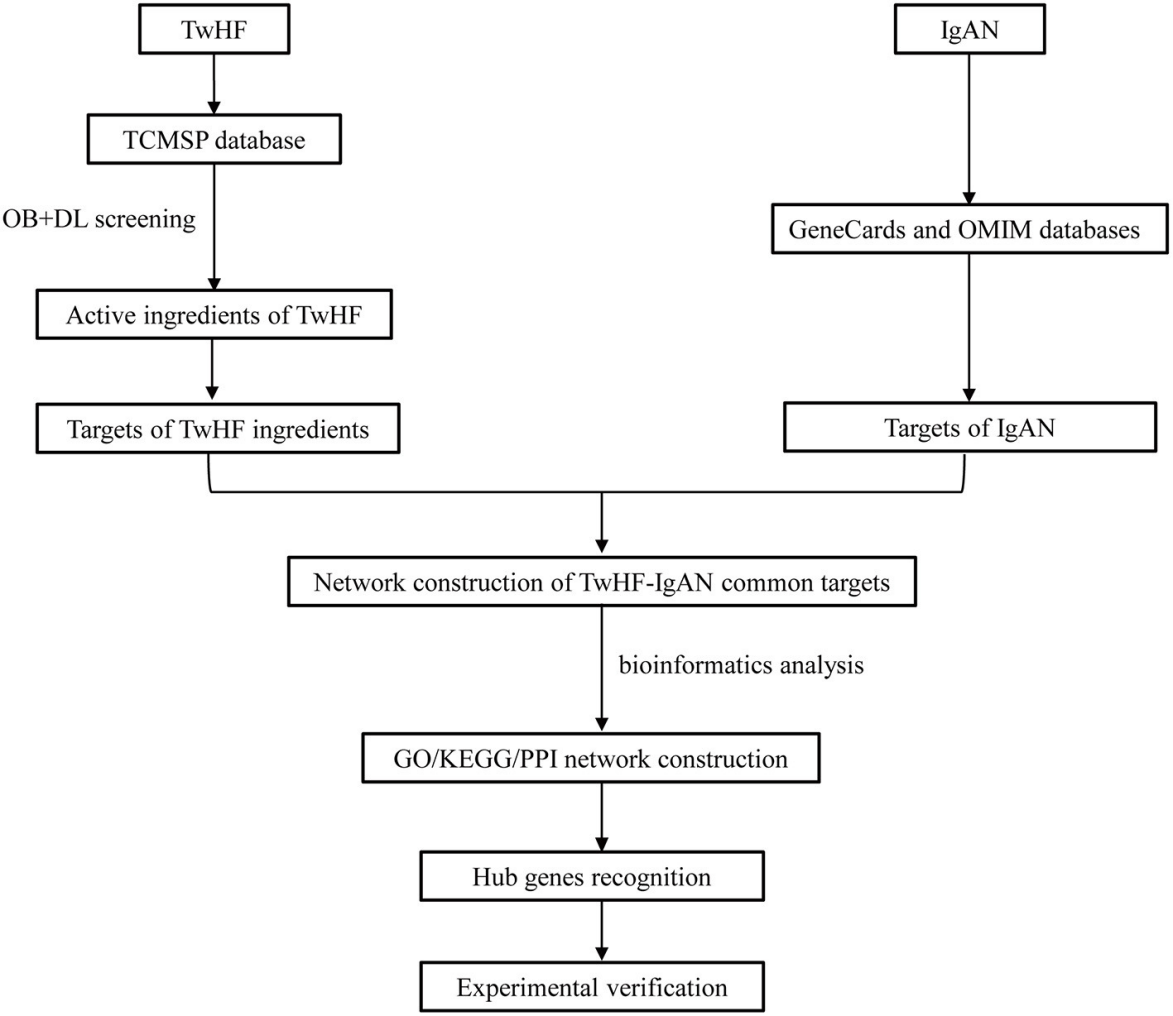
A flow chart exploring TwHF against IgAN based on network pharmacology.

**Figure 2:**
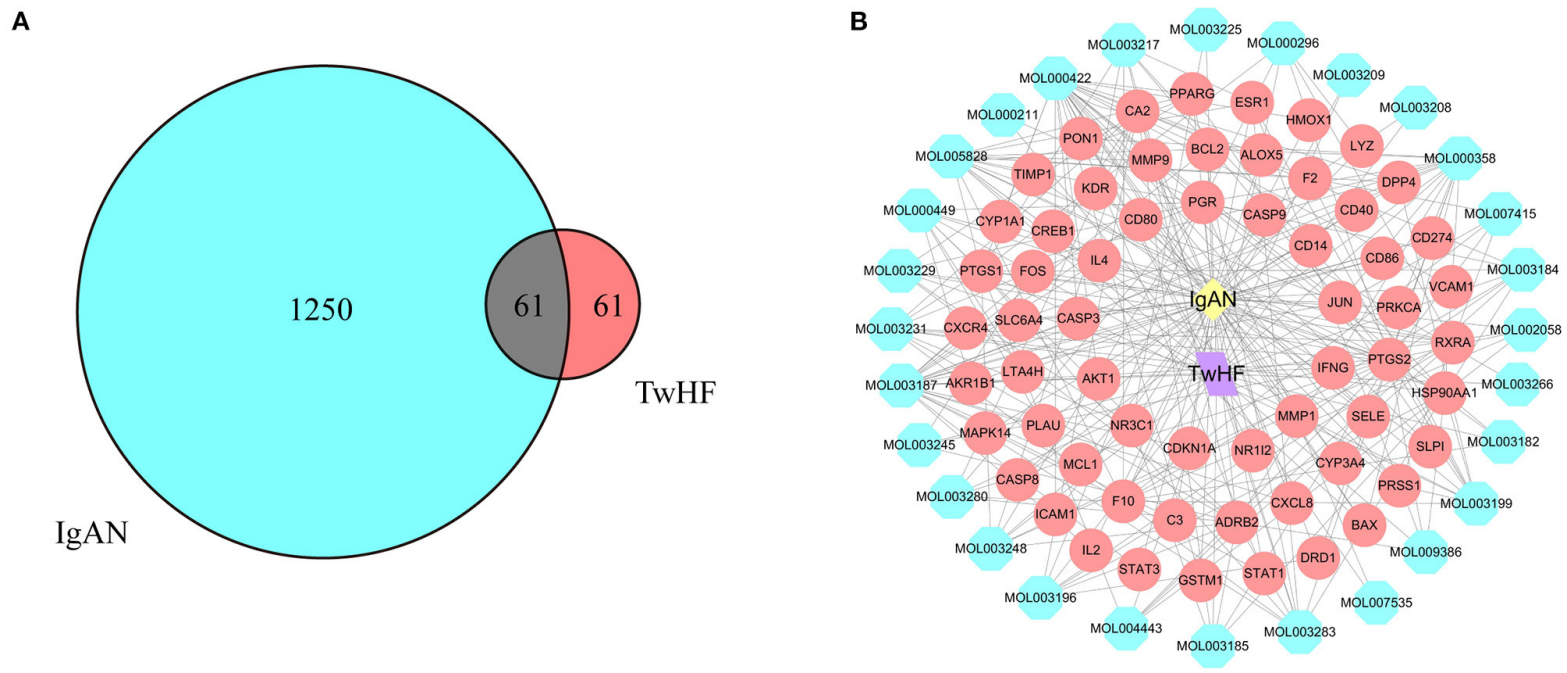
Network of potential targets between IgAN and TwHF ingredients. **(A)** Overlapping target genes between IgAN and TwHF. **(B)** Network of putative targets. The yellow and purple nodes stand for IgAN and TwHF, respectively. The blue nodes represent the active components of TwHF, and the red nodes represent the corresponding targets. The lines stand for interactions.

The flow chart of the study was shown in Figure 1. The active chemical components of TwHF were selected *via* the Traditional Chinese Medicine Systems Pharmacology Database (TCMSP, http://tcmspw.com/tcmsp.php). With oral bioavailability (OB) ≥ 30% and the drug-likeness (DL) ≥0.18, 51 candidate compounds (Supplementary Table 1) and 122 corresponding targets were collected for further analysis. To visualize the interaction between TwHF targets and IgAN, 1311 potential IgAN targets were obtained using the GeneCards (https://www.genecards.org) and Online Mendelian Inheritance in Man (OMIM, https://www.omim.org) databases. The common targets of TwHF ingredients and IgAN were screened (Figure 2A). By mapping 61 target genes to TwHF and IgAN, a network that comprises 91 nodes and 282 edges was established (Figure 2B).

### Functional Enrichment Analysis

**Figure 3:**
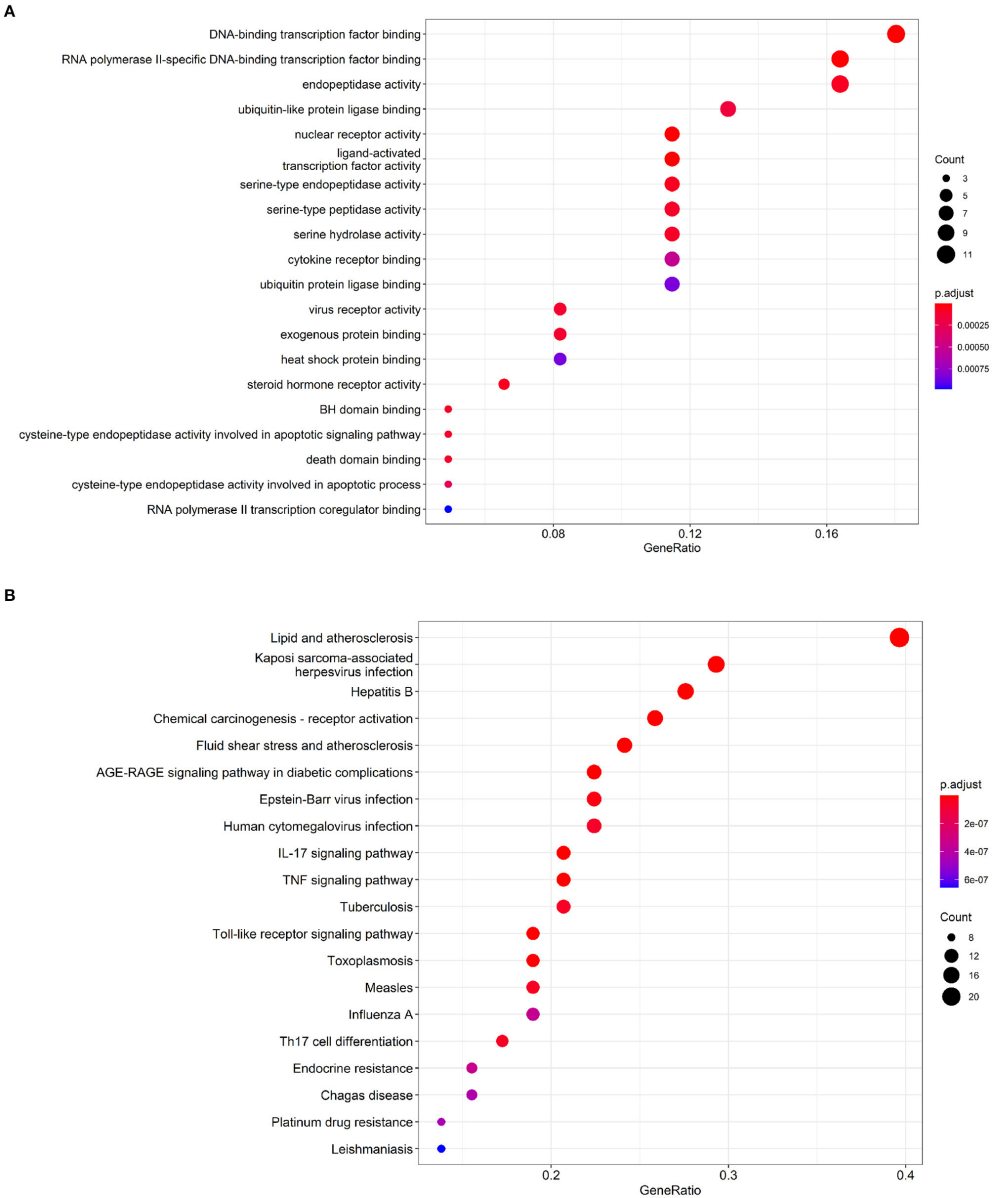
Functional enrichment analysis of the IgAN-TwHF ingredients common targets. **(A)** Top 20 GO terms and **(B)** Top 20 enriched KEGG pathways of targets. The color depth of the nodes refers to the adj p-value. The size of the nodes refers to the number of genes.

To identify comprehensive information on biological processes, cellular components, and molecular functions gene function, GO and KEGG pathway analyses of common targets were performed *via* clusterProfiler package in R software. Values of *p* < 0.05 and *q* < 0.05 were set as the threshold. The top 20 GO terms with the highest degree of enrichment were shown in Figure 3A. GO terms mainly concentrated on DNA-binding transcription factor binding, RNA polymerase II-specific DNA-binding transcription factor binding, and endopeptidase activity. Figure 3B presented 20 enriched KEGG pathways of the common targets, lipid and atherosclerosis pathway, Kaposi sarcoma-associated herpesvirus infection, and hepatitis B were identified as the top three significantly relevant signal pathways.

### PPI Network Construction and key Genes Recognition

**Figure 4:**
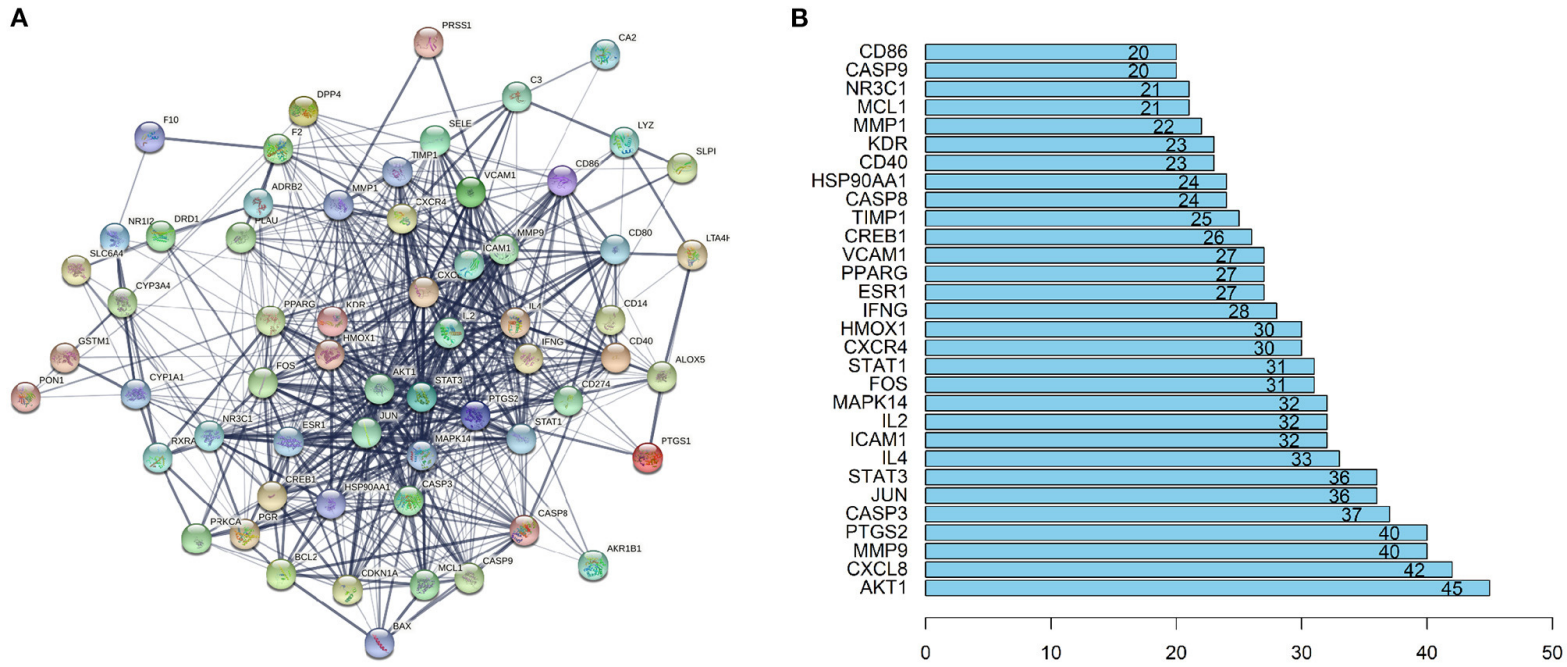
PPI network and the key genes of the IgAN-TwHF ingredients common targets. **(A)** The PPI network of targets was constructed using string. **(B)** The Top 30 key genes were obtained from the PPI network. The numbers on the bar represent the number of related nodes for each gene.

The STRING database (https://string-db.org) was used to explore potential interactions between targets, and visualization of protein-protein interaction (PPI) network was shown in Figure 4A. 592 interactions were calculated by R software, the top 30 candidate hub genes which may play a central role in this network were identified in Figure 4B. Targets with higher degrees including *AKT1, CXCL8, MMP9, PTGS2, CASP3*, and *JUN* were identified as key genes.

### Targets Verification

*Tripterygium wilfordii* polyglycosides (TGT) is widely used in clinical practice on IgAN in China, with triptolide as the effective non-steroidal immunosuppressive ingredient (9). Considering we have previously found an important role of JUN signaling in IgAN mesangial cells, we determined to verify *JUN* (also known as *c-JUN*) as the target of TGT or triptolide treatment both *in vivo* and *in vitro*.

**Figure 5:**
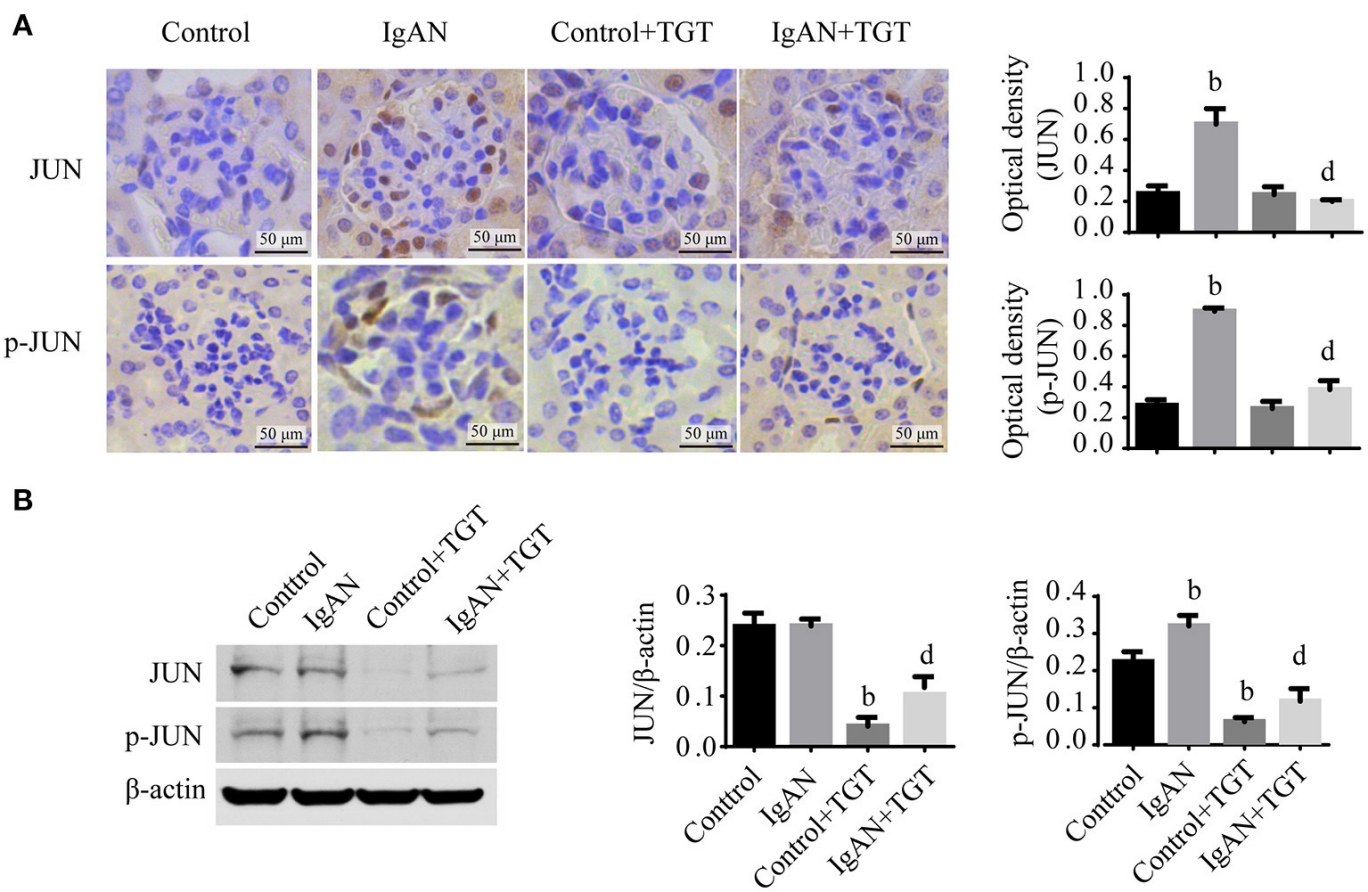
TGT downregulated JUN signaling in the kidney of IgAN mice. **(A)** Representative images and quantitative analysis of glomerular immunohistostaining with JUN and p-JUN in TGT treated mice or control mice. The average optical density was analyzed by ImageJ software. **(B)** Western blot analysis for JUN, p-JUN expressions in IgAN. Data are presented as mean ± *SD* of three independent experiments. ^b^*p* < 0.01 vs. control group, ^d^*p* < 0.01 vs. IgAN group.

The immunohistochemical results of p-JUN were significantly increased in IgAN mice compared with controls, while treatment with TGT significantly decreased p-JUN expression (Figure 5A). Western blot analysis further confirmed that TGT suppressed phosphorylation of JUN in IgAN (Figure 5B).

**Figure 6:**
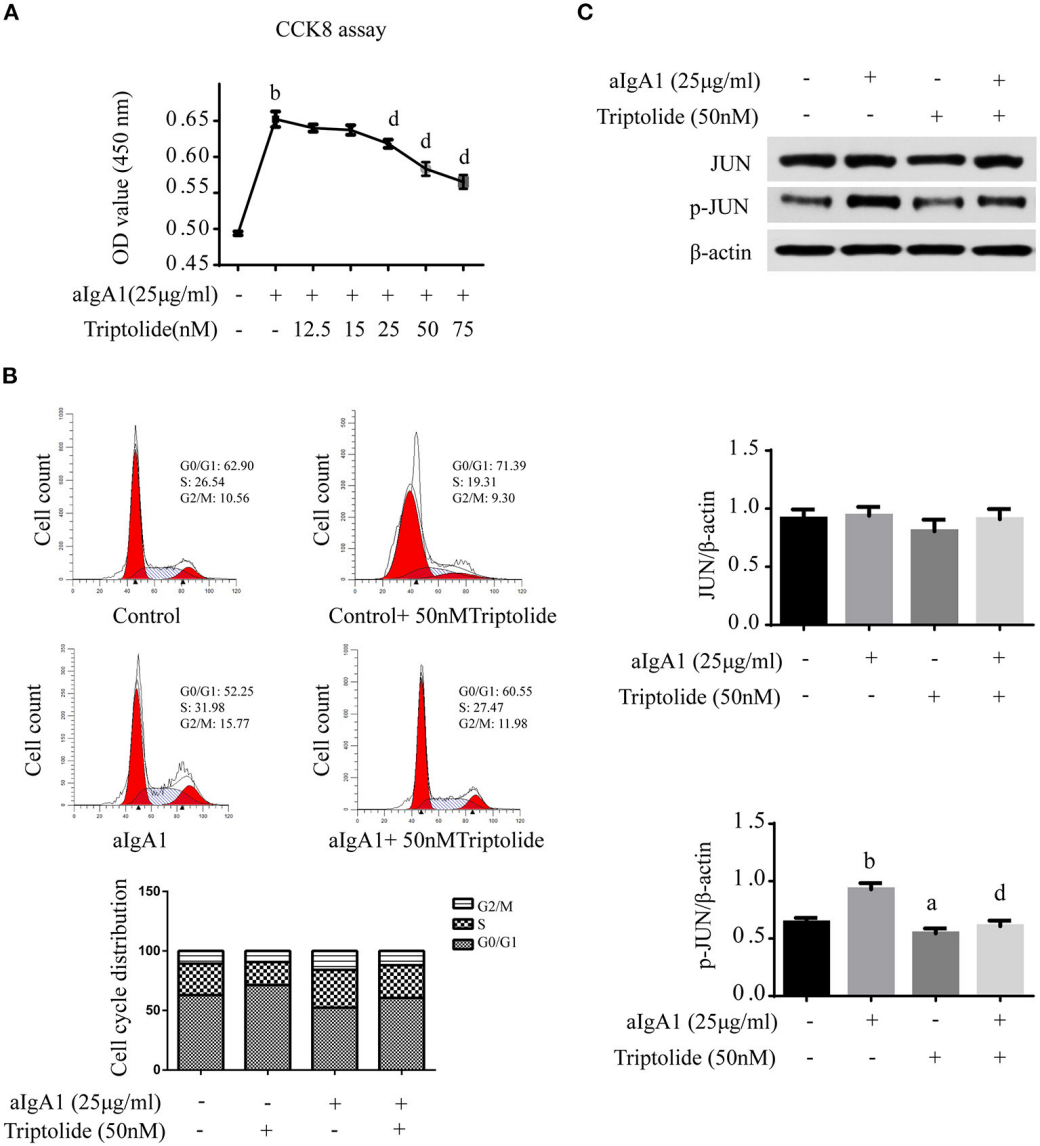
Triptolide inhibited HMCs proliferation and reduced phosphorylation of JUN signaling. **(A)** The proliferation of HMCs with different concentrations of triptolide intervention was assessed by CCK8 assay. **(B)** The cell cycle of HMCs treated with 25 μg/ml aIgA1 and 50 nM triptolide was detected by flow cytometry. **(C)** Immunoblot analyses and quantitative determination of the protein levels of JUN, p-JUN in HMCs. Data are the mean ± SD of three independent experiments. ^a^*p* < 0.05, ^b^*p* < 0.01 vs. no treatment group, ^d^*p* < 0.01 vs. only aIgA1-treated group.

Since mesangial cell proliferation is a prominent pathological feature of IgAN, *in vitro*, we investigated the effect of TwHF on HMCs. Moreover, 25 μg/ml aIgA1-stimulated HMCs were used to mimic the IgAN HMCs model, as previously described (21). The CCK8 assay showed that triptolide diminished cell proliferation in a concentration-dependent manner within 25–75 nM after 24 h treatment (Figure 6A), and there was no significant cell morphology alteration. In addition, flow cytometry was performed to detect cell cycle. Results showed that aIgA1 increased the proportion of cells in S and G2/M phase after 24 h incubation, with the application of 50 nM triptolide, more cells were arrested in G0/G1 phase (Figure 6B).

Finally, we examined the effect of triptolide on the JUN signaling in IgAN. Western blot analysis showed that 50 nM triptolide efficiently suppressed the phosphorylation of JUN (Figure 6C).

## Discussion

The first description of IgA nephropathy was proposed by Jean Berger in 1968, thus it is also named Berger's s disease (22). At present, there is still no specific treatment for IgAN. Supportive care is the basis including angiotensin-converting enzyme inhibitors (ACEI) or angiotensin receptor blockers (ARB), adequate blood pressure control, low-salt diet, and aerobic exercise. TwHF contains several active ingredients which affect multiple targets and pathways in the progression of IgAN and has been used as an adjuvant on IgAN treatment (11). A wide spectrum of biological activities about TwHF including anti-inflammatory, immunosuppressive, antioxidation, and antifibrosis has been demonstrated (9, 23–26). Triptolide, a diterpenoid trioxide, is the most abundant and pharmacologically active of the metabolites found in TwHF extracts and is primarily responsible for the anti-inflammatory and immunosuppressive effects of TwHF preparation (27, 28). Evidence has emerged to elucidate the biological effects of triptolide or its pharmaceutical preparation TGT in IgAN. TGT was reported to inhibit the secretion of serum inflammatory factor TNF-α, IL-6, and TGF-β1 in IgAN (29). Our team previously showed that triptolide decrease pro-inflammatory cytokines IL-1β and IL-18 in serum *via* the NLRP3/TLR4 pathway, and reduced the deposition of immune complexes-induced mesangial cell proliferation in IgAN rats (30). The early study was also observed that triptolide could effectively up-regulate the expression of nephrin protein, protect podocytes and reduce urinary protein in the rat model of IgAN (31). All the above evidence proves that TwHF has great benefits on IgAN, however, the pharmacological mechanisms of TwHF associated with IgAN only focus on a single chemical molecule. Here, we performed a comprehensive and systematic evaluation of the molecular mechanisms of TwHF on IgAN and highlighted new perspectives of TwHF for delaying IgAN progression and strengthening the therapeutic theory.

In this study, 51 active ingredients were selected from TwHF and 61 common targets were matched between the components of TwHF and IgAN based on network pharmacology. Functional enrichment analysis suggested that TwHF mainly interfere with the binding of transcription factors to regulatory regions of genes and induce or inhibit its gene expression. According to the PPI network, candidate key genes of TwHF against IgAN were identified as *AKT1, CXCL8, MMP9, PTGS2, CASP3*, and *JUN*. AKT and CXCL8 were reported to be involved in the inflammatory response and were activated during renal injury (32–35), polymorphism of *CXCL8* was associated with increased susceptibility to IgAN (36). Studies also suggested that triptolide could attenuate renal tubular epithelial-mesenchymal transition *via* AKT signaling in diabetic kidney disease (37). In addition, TwHF extracts contained novel inhibitors of MMP including MMP9 thus might have therapeutic potential in arthritis and other conditions associated with increased MMPs like IgAN (38, 39). CASP3 is known as a representative effector of apoptosis, cleaved caspase-3 active fragment could mediate apoptosis in endothelial cells (40). PTGS2 also known as cyclooxygenase 2 (COX2), is the key enzyme in prostaglandin biosynthesis, and is responsible for the prostanoid biosynthesis involved in inflammation and mitogenesis. COX-derived prostanoids appear to be involved in the pathogenesis of diabetic nephropathy (41), and a recent study regarded prostaglandins as potential targets for the treatment of polycystic kidney disease (42). JUN is a positive regulator of cell proliferation which promotes cell proliferation by accelerating G1-S cell cycle progression (43–45). It could bind with FOS to form AP-1 (Activator protein-1) early transcription response factor and mediate many biological functions (46, 47). Our previous study confirmed the pivotal role of c-Jun N-terminal protein kinase (JNK)/JUN in IgAN mesangial cell proliferation and renal lesions, activation of c-Jun promoted cyclinD1 and proliferating cell nuclear antigen (PCNA) expression, accelerating the proliferation of IgAN mesangial cells (19). Considering that JUN ranked among the best in the results of functional enrichment analysis and PPI network, we thus verified whether TwHF could attenuate IgAN renal injury by regulating JUN. As shown in our study, TGT treatment could reduce the proliferation of mesangial cells and inhibit p-JUN signaling. *In vitro*, JUN phosphorylation was enhanced after aIgA1 treatment, while was abolished when triptolide was added. Cell cycle detection showed that the HMCs were arrested in the G1 phase after triptolide intervention.

JUN is generally considered to be essential for many biological functions especially in the progression of the G1 phase of the cell cycle (48, 49). Cells lacking JUN increased p53 (cell cycle arrest inducer) and p21 (cell cycle protease inhibitor 1, p53 target gene) expressions while overexpressing JUN reversed the levels of p53 and p21 and showed accelerated cell cycle transition as well as cell proliferation (50, 51). In addition, JUN could regulate the transcription of Rb kinase cyclinD1 to promote cell cycle transition (52). JUN signaling was also reported to be associated with renal interstitial fibrosis (53, 54). Here, we verified JUN as potential therapeutic targets for TwHF treatment based on network pharmacology tools, further research is required to detect the variety of changes occurring to cellular processes and downstream of JUN signaling with/without administration of TwHF following knockdown of JUN expression.

## Data Availability Statement

The original contributions presented in the study are included in the article/Supplementary Material, further inquiries can be directed to the corresponding author/s.

## Ethics Statement

The studies involving human participants were reviewed and approved by Ethics Committee of the Second Xiangya Hospital of Central South University. The patients/participants provided their written informed consent to participate in this study. The animal study was reviewed and approved by Animal Care Ethics Committee of Xiangya Medical School, Central South University.

## Author Contributions

MX contributed to the study design, experiments, and draft of the manuscript. DL, HL, and JZ contributed to data collection and statistical analysis/interpretation. CT, GC, and YL contributed to review and editing. HL gave the final approval for the article to be published. All authors have read and approved the final manuscript.

## Funding

This work was supported by the National Natural Science Foundation of China (82070737 and 81770714), Scientific Research Key Project of Hunan Traditional Chinese Medicine Administration (2021040), and PRO∙Run Fund of the Nephrology Group of CEBM (KYS2021-03-02-7).

## Conflict of Interest

The authors declare that the research was conducted in the absence of any commercial or financial relationships that could be construed as a potential conflict of interest.

## Publisher's Note

All claims expressed in this article are solely those of the authors and do not necessarily represent those of their affiliated organizations, or those of the publisher, the editors and the reviewers. Any product that may be evaluated in this article, or claim that may be made by its manufacturer, is not guaranteed or endorsed by the publisher.
