# Supplementary material for: Based on Network Pharmacology Tools to Investigate the Mechanism of Tripterygium wilfordii Against IgA Nephropathy
Source: Front Med (Lausanne). 2021 Dec 15;8:794962. doi: 10.3389/fmed.2021.794962 (PMC8715946; doi:10.3389/fmed.2021.794962)
Supplement: Supplementary file 1 [file Table_1.DOCX]

Table S1. Basic information of active components in TwHF.

| Mol ID | Molecule Name | MW | OB(%) | DL |
| --- | --- | --- | --- | --- |
| MOL000211 | Mairin | 456.78 | 55.37707338 | 0.7761 |
| MOL000296 | hederagenin | 414.79 | 36.91390583 | 0.7507 |
| MOL000358 | beta-sitosterol | 414.79 | 36.91390583 | 0.7512 |
| MOL000422 | kaempferol | 286.25 | 41.88224954 | 0.2407 |
| MOL000449 | Stigmasterol | 412.77 | 43.82985158 | 0.7567 |
| MOL002058 | 40957-99-1 | 388.45 | 57.20447445 | 0.6187 |
| MOL003182 | (+)-Medioresinol di-O-beta-D-glucopyranoside_qt | 388.45 | 60.69319498 | 0.6188 |
| MOL003184 | 81827-74-9 | 342.47 | 45.41820853 | 0.5331 |
| MOL003185 | (1R,4aR,10aS)-5-hydroxy-1-(hydroxymethyl)-7-isopropyl-8-methoxy-1,4a-dimethyl-4,9,10,10a-tetrahydro-3H-phenanthren-2-one | 346.51 | 48.84498719 | 0.3833 |
| MOL003187 | triptolide | 360.44 | 51.28850027 | 0.6773 |
| MOL003188 | Tripchlorolide | 396.9 | 78.71907822 | 0.7184 |
| MOL003189 | WILFORLIDE A | 486.81 | 35.6639958 | 0.7176 |
| MOL003192 | Triptonide | 344.39 | 67.65501583 | 0.7039 |
| MOL003196 | Tryptophenolide | 312.44 | 48.49645571 | 0.4442 |
| MOL003198 | 5 alpha-Benzoyl-4 alpha-hydroxy-1 beta,8 alpha-dinicotinoyl-dihydro-agarofuran | 600.72 | 35.26387229 | 0.7243 |
| MOL003199 | 5,8-Dihydroxy-7-(4-hydroxy-5-methyl-coumarin-3)-coumarin | 352.31 | 61.84861803 | 0.5374 |
| MOL003206 | Canin | 278.33 | 77.4069579 | 0.3322 |
| MOL003208 | Celafurine | 369.51 | 72.94407141 | 0.4406 |
| MOL003209 | Celallocinnine | 405.59 | 83.47184251 | 0.5905 |
| MOL003210 | Celapanine | 569.66 | 30.17588389 | 0.8233 |
| MOL003211 | Celaxanthin | 550.94 | 47.37181232 | 0.5776 |
| MOL003217 | Isoxanthohumol | 354.43 | 56.81068162 | 0.3895 |
| MOL003222 | Salazinic acid | 402.33 | 36.34126161 | 0.7627 |
| MOL003224 | Tripdiotolnide | 360.44 | 56.3975191 | 0.6691 |
| MOL003225 | Hypodiolide A | 318.5 | 76.12897807 | 0.4937 |
| MOL003229 | Triptinin B | 314.46 | 34.73361611 | 0.3236 |
| MOL003231 | Triptoditerpenic acid B | 328.49 | 40.01842572 | 0.3567 |
| MOL003232 | Triptofordin B1 | 478.63 | 39.55214125 | 0.84 |
| MOL003233 | Triptofordin B2 | 608.69 | 107.7109856 | 0.7552 |
| MOL003234 | Triptofordin C2 | 610.71 | 30.1626851 | 0.7575 |
| MOL003235 | Triptofordin D1 | 606.72 | 31.99926478 | 0.755 |
| MOL003236 | Triptofordin D2 | 650.78 | 30.37595914 | 0.6865 |
| MOL003238 | Triptofordin F1 | 694.79 | 33.90626875 | 0.6013 |
| MOL003239 | Triptofordin F2 | 668.75 | 33.61785503 | 0.6672 |
| MOL003241 | Triptofordin F4 | 652.75 | 31.37039593 | 0.6735 |
| MOL003242 | Triptofordinine A2 | 741.85 | 30.77868068 | 0.475 |
| MOL003244 | Triptonide | 358.42 | 68.44945585 | 0.6787 |
| MOL003245 | Triptonoditerpenic acid | 344.49 | 42.5608213 | 0.3923 |
| MOL003248 | Triptonoterpene | 300.48 | 48.56909374 | 0.2831 |
| MOL003266 | 21-Hydroxy-30-norhopan-22-one | 428.77 | 34.10829417 | 0.7701 |
| MOL003267 | Wilformine | 805.86 | 46.31588853 | 0.2012 |
| MOL003278 | salaspermic acid | 472.78 | 32.19066882 | 0.6313 |
| MOL003279 | 99694-86-7 | 376.44 | 75.23328061 | 0.6618 |
| MOL003280 | TRIPTONOLIDE | 326.42 | 49.50852641 | 0.4879 |
| MOL003283 | (2R,3R,4S)-4-(4-hydroxy-3-methoxy-phenyl)-7-methoxy-2,3-dimethylol-tetralin-6-ol | 360.44 | 66.51046743 | 0.3887 |
| MOL004443 | Zhebeiresinol | 280.3 | 58.72053449 | 0.1938 |
| MOL005828 | nobiletin | 402.43 | 61.66943932 | 0.5165 |
| MOL007415 | [(2S)-2-[[(2S)-2-(benzoylamino)-3-phenylpropanoyl]amino]-3-phenylpropyl] acetate | 444.57 | 58.01629624 | 0.5196 |
| MOL007535 | (5S,8S,9S,10R,13R,14S,17R)-17-[(1R,4R)-4-ethyl-1,5-dimethylhexyl]-10,13-dimethyl-2,4,5,7,8,9,11,12,14,15,16,17-dodecahydro-1H-cyclopenta[a]phenanthrene-3,6-dione | 428.77 | 33.1153996 | 0.788 |
| MOL009386 | 3,3'-bis-(3,4-dihydro-4-hydroxy-6-methoxy)-2H-1-benzopyran | 358.42 | 52.10867137 | 0.544 |
| MOL011169 | Peroxyergosterol | 428.72 | 44.39151838 | 0.82 |
